# Research Strategies and Methods of Hydrogels for Antitumor Drug Delivery

**DOI:** 10.3390/biomedicines13081899

**Published:** 2025-08-04

**Authors:** Tianjiao Zeng, Lusi Chen, Toru Yoshitomi, Naoki Kawazoe, Yingnan Yang, Guoping Chen

**Affiliations:** 1 Research Center for Macromolecules and Biomaterials, National Institute for Materials Science, Namiki 1-1, Tsukuba 305-0044, Ibaraki, Japan; zeng.tianjiao@nims.go.jp (T.Z.); lschen.1214@gmail.com (L.C.); yoshitomi.toru@nims.go.jp (T.Y.); kawazoe.naoki@nims.go.jp (N.K.); 2 Graduate School of Science and Technology, University of Tsukuba, Tsukuba 305-8577, Ibaraki, Japan; 3 Graduate School of Life and Environment Science, University of Tsukuba, 1-1-1 Tennodai, Tsukuba 305-8572, Ibaraki, Japan; yo.innan.fu@u.tsukuba.ac.jp

**Keywords:** hydrogel, drug delivery system, antitumor therapy, combination therapy

## Abstract

Tumor treatments have substantially advanced through various approaches, including chemotherapy, radiotherapy, immunotherapy, and gene therapy. However, efficient treatment necessitates overcoming physiological barriers that impede the delivery of therapeutic agents to target sites. Drug delivery systems (DDSs) are a prominent research area, particularly in tumor therapy. This review provides a comprehensive overview of hydrogel-based DDSs for tumor treatment, focusing on the strategies and designs of DDSs based on the unique pathophysiological characteristics of tumors. The design and preparation of hydrogel systems for DDSs are summarized and highlighted. The challenges and opportunities for translating hydrogel-based DDSs into clinical applications are discussed.

## 1. Introduction

Drug delivery systems (DDSs) have attracted considerable attention, particularly in the context of antitumor treatments. A DDS is a formulation or device that enables the introduction of therapeutic substances into the body and improves treatment efficiency and safety by controlling the rate, timing, and location of drug release [1]. The sustained drug mechanism of DDSs is more conducive to eliminating tumor cells compared with that using free drug administration [2,3,4]. Furthermore, during antitumor therapy, the DDS improves local drug accumulation, which enhances therapeutic efficacy and reduces off-target toxicity to normal cells, thus alleviating safety issues [5,6].

Currently, a variety of carriers are used in DDSs for tumor therapy, including liposomes, nanoparticles (NPs), and hydrogels [7,8,9]. Among them, liposomes have achieved clinical success and several formulations have already been approved for use. For example, Doxil was the first liposomal product approved by the FDA for ovarian tumor treatment in 1995. More recently, Vyxeos is a new liposomal product that combines two currently used chemotherapies (daunorubicin and cytarabine) for leukemia treatment [10]. Other nanocarriers, such as dendrimers and inorganic NPs, have also been explored for DDS applications [11,12]. Despite the differences in their mechanisms and applications, most DDSs focus on three primary goals: achieving controlled drug release, targeting specific release sites, and maintaining the therapeutic potency of drugs. However, liposomes and NPs have limitations, including low drug entrapment efficiency, endosomal entrapment, and rapid clearance by the mononuclear phagocyte system, which ultimately diminishes their antitumor efficacy [13,14,15,16]. Therefore, alternative biomaterials capable of enabling controlled drug release are required to overcome these drawbacks.

Hydrogels, a class of water-swollen polymer networks composed of macromolecules crosslinked either physically or chemically, are promising candidates for DDSs. Hydrogels have received substantial attention as drug delivery devices in medical applications, especially in antitumor treatment, owing to their high water content, excellent biocompatibility, combinatorial optimization potential, and adaptable structure.

For example, hydrophilic drugs, such as vitamin C (Vit C), often face challenges due to rapid excretion, limiting their therapeutic effectiveness. To address this issue, Zhang et al. developed amphiphilic vitamin C self-assembled nanofiber hydrogels to achieve controlled release. By chemically modifying Vit C with a hydrophobic alkyl chain linked via an ester bond that is enzymatically degradable in vivo, hydrogels are formed through hydrogen bonding and hydrophobic interactions and can be used as Vit C depots. Approximately 75% of Vit C was released over 10 days, demonstrating a controllable release profile and improved antitumor effects [17]. In another example, alginate–pectin hydrogels were prepared using ionic crosslinking for the oral delivery of resveratrol (RES) to the intestines. Alginate hydrogels remained stable in acidic environments but degraded under alkaline conditions, whereas pectin enhanced the mechanical strength of the hydrogels. This approach protects RES from stomach degradation, enables targeted delivery to the intestine, and improves therapeutic outcomes [18].

Moreover, hydrogels combined with liposomes can markedly enhance drug delivery efficiency. Li et al. developed a multilevel DDS platform in which drugs were encapsulated in liposomes and subsequently coated with thiolate chitosan to form a liposomal hydrogel. Hydrophobic curcumin was loaded into liposomes to enhance its solubility, enabling localized drug delivery. This system demonstrated superior antitumor efficacy compared with that of free drugs or liposomal drugs alone, effectively inhibiting tumor recurrence and promoting tissue repair [19]. Hydrogels have been extensively studied as drug delivery devices for disease treatment.

**Figure 1:**
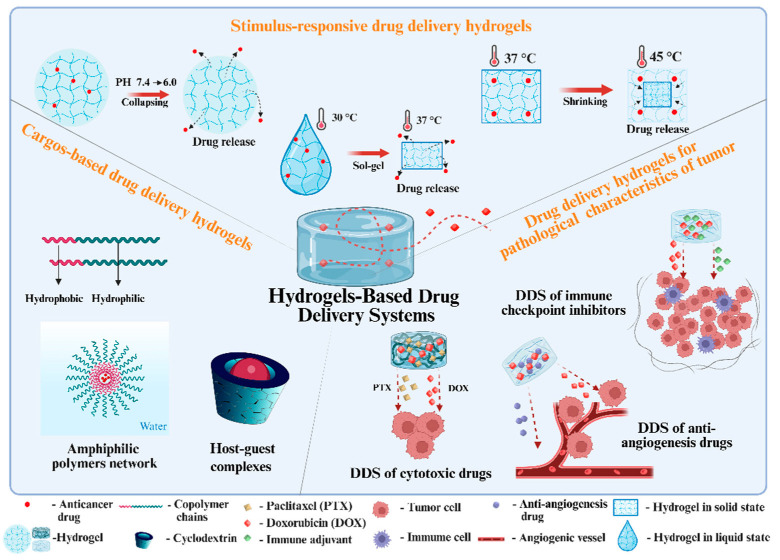
Schematic illustration of various strategies used to prepare hydrogel-based drug delivery systems (DDSs) for antitumor therapy. The illustration was created using BioRender, https://app.biorender.com/ (accessed on 27 June 2025).

Despite extensive research on hydrogel-based DDSs, most existing reviews have focused on their fundamental properties and general applications [20,21,22]. Few reviews have focused on the strategies and methods for designing hydrogels specifically for antitumor drug delivery. Hence, this review summarizes the strategies and preparation methods for hydrogels used in antitumor therapy, with an emphasis on common approaches for evaluating hydrogel-based DDSs both in vitro and in vivo (Figure 1).

## 2. Design of Hydrogel-Based DDSs

Hydrogel materials are broadly categorized as naturally derived, semisynthetic, and synthetic polymers [23]. Naturally derived polymers such as collagen, gelatin, and fibrin exhibit advantageous properties, including biodegradability, low cytotoxicity, and a minimal immune response in vivo. Additionally, many naturally derived polymers facilitate cellular adhesion, making them suitable for applications in tissue engineering and localized DDSs [24,25,26]. Commonly used synthetic polymers exhibit good mechanical features, plasticity, and variable properties for on-demand applications [27,28]. Following this development [29], polymers frequently used to prepare hydrogel-based DDSs are summarized in Table 1.

**Table 1:** Polymers and preparation methods for hydrogel-based DDSs after 2019.

Polymers		Loading Drugs	Preparing Method	Degradation and Stability	Sterilization Method	In Vitro/In Vivo Analysis	Biocompatibility	Reference
Naturally derived polymers	Gelatin and alginate	Doxorubicin (DOX)	Chemically crosslinking by “Shift-Base” condensation reaction	/	No description while Schiff base has part of antibacterial effect	In vitro	Good biocompatibility (less than 10% decrease in cell viability of no-drug-loading hydrogel)	[30]
	Bisphosphonate-functionalized hyaluronic acid	DOX	Chemical interaction of bisphosphonate–zinc	60% mass decrease in drug-loading hydrogel after 316 h in medium	/	In vitro and in vivo	Good biocompatibility (less than 5% decrease in cell viability of no-drug-loading hydrogel)	[31]
	Alginate sodium carboxymethyl cellulose	Methotrexate (MTX) and aspirin (AP)	Physical crosslinking	42% mass decrease after 8 days in PBS	/	In vitro	Good biocompatibility (less than 5% decrease in cell viability of no-drug-loading hydrogel)	[32]
	Alginate and magnetic hydroxyapatite	5-fluorouracil (5-FU)	Physical crosslinking	Increasing light and temperature reduces the storage stability	Filtration	In vitro	Good biocompatibility (less than 15% decrease in cell viability of no-drug-loading hydrogel)	[33]
Synthetic polymers	Poly (D, L-lactide-co-glycolide)-poly(ethylene-glycol)-poly (D, L-lactide-co-glycolide), beta-cyclodextrin	DOX and curcumin	Physical crosslinking	13.3% mass decrease after 24 h in PBS	/	In vitro and in vivo	Good biocompatibility (less than 10% decrease in cell viability of no-drug-loading hydrogel)	[34]
	Methacrylic acid (MAA)	Camptothecin (CPT)	Chemically crosslinking through disulfide linkage between CPT and MAA	Stable in normal physiological environment; quickly shrinks to a smaller volume in low pH environment of tumor tissue or tumor cells	/	In vitro and in vivo	Good biocompatibility (less than 20% decrease in cell viability of no-drug-loading hydrogel)	[35]
	Polyethylene glycol (PEG)	DOX and curcumin	Chemically crosslinking	Instability	/	In vitro	Good biocompatibility (less than 10% decrease in cell viability of no-drug-loading hydrogel)	[36]
	Polyacrylamide (PAM) and carbon nanotube	DOX	Physically crosslinking by hydrogen bond	Stable in PBS after 80 h	/	In vitro	Good biocompatibility (less than 20% decrease in cell viability of no-drug-loading hydrogel)	[37]
Combination and/or modified polymers	Chitosan, polypyrrole	DOX	Chemically crosslinking by “Shift-Base” condensation reaction	Stable in PBS (both pH 7.4 and 6.5) after 7 days	/	In vitro and in vivo	Good biocompatibility (less than 3% decrease in cell viability of no-drug-loading hydrogel)	[38]
	PEG-modified bovine serum albumin	Paclitaxel (PTX)	Physical crosslinking of PEG-BSA	46% mass loss in PBS after 50 days. Degraded completely within 200 days in vivo	/	In vitro and in vivo	No toxicity of hydrogel	[39]
	Carboxymethyl arabinoxylan	5-FU	Physically crosslinking	5 to 15% mass decrease after 7 days in PBS, 37 °C	The nanocomposite hydrogel has antibacterial activity	In vitro	Degradation leads to cell death	[40]
	Carboxymethyl chitosan and polyvinyl alcohol	Oxaliplatin	Chemically crosslinking by free-radical polymerization	/	/	In vitro and in vivo	Good biocompatibility by acute oral toxicity assay	[41]
	Ultrasmall peptide	DOX	Dimerization of the PyKC peptide	High stability after 18 days in vivo	/	In vitro and in vivo	Good biocompatibility (less than 5% decrease in cell viability of no-drug-loading hydrogel)	[42]
	Thiol-modified hyaluronic acid (HASH) and vinyl sulfone-modified β-cyclodextrin	DOX	Chemically crosslinking of a “click reaction” between thiol and vinyl sulfone groups	Keep stable after 15 days in PBS while degraded after 5 days in enzymatic conditions	/	In vitro	Good biocompatibility	[43]
	N-isopropylacrylamide (NIPAAm) and maleic anhydride (MA) copolymer (poly (NIPAAm-co-MA)) chitosan	MTX and curcumin	Physical crosslinking	Over 80% weight loss after 28 days in PBS	/	In vitro	Good biocompatibility (less than 10% decrease in cell viability of no-drug-loading hydrogel)	[44]

Although simple polymer hydrogels have been successfully used in DDSs, hybrid hydrogels composed of combinations of different polymers provide enhanced versatility [45]. Regardless of the type of polymer used to prepare the hydrogels, environment-responsive functionalities and cargo-specific designs are the two key considerations that dictate polymer selection and modification to achieve efficient and safe drug delivery in clinical applications.

### 2.1. Stimulus-Responsive Drug Delivery Hydrogels

The tumor microenvironment (TME) significantly differs from normal tissues and is characterized by unique features, such as low pH, hypoxia, thermal sensitivity, and even different stiffness or viscosity of the extracellular matrix (ECM) [46]. These distinct properties of the TME not only influence the therapeutic response and clinical outcome [47] but also provide opportunities to design materials that specifically target the TME and physiological conditions, thereby improving safety and therapeutic efficacy [48]. Accordingly, stimuli-responsive materials have been extensively studied for DDSs.

Using the acidic pH of the TME to develop pH-responsive hydrogels is one of the most widely studied strategies (Table 2). The extracellular pH in tumor sites typically ranges from 6.5 to 7.0, caused by the high metabolic rate of tumors [49], whereas the pH value of normal tissues maintains a pH of approximately 7.4 [50]. pH-sensitive hydrogels exploit this difference to enhance drug release, specifically at tumor sites, thereby improving localized drug concentration and minimizing systemic toxicity. For instance, Qu et al. developed a hydrogel for the delivery of doxorubicin (DOX), a commonly used first-line antitumor agent. The hydrogel was synthesized using N-carboxyethyl chitosan (CEC) via the Michael reaction in an aqueous solution, combined with dibenzaldehyde-terminated poly (ethylene glycol) (PEGDA). Under acidic conditions, the protonated and positively charged amino groups of chitosan weaken the Schiff base bonds between -NH_2_ and -CHO, accelerating hydrogel degradation and enabling faster drug release in an acidic environment than in a neutral environment [51]. Qing et al. designed a hydrogel using dopamine, carboxymethyl cellulose (CMC), and hydroxyethyl cellulose to deliver the antibacterial drug, ciprofloxacin (CIP). In this system, the hydrolysis of the amide bond between CMC and CIP is the primary mechanism controlling drug release and is highly sensitive to pH changes [52]. Mukherjee et al. demonstrated that acidic environments not only increased the swelling ratio of the hydrogel but also facilitated the breakage and degradation of ester bonds within the polymer matrix, thus enhancing drug release [53].

The design of pH-responsive hydrogels typically aims to modulate the drug release profile by accelerating hydrogel degradation or weakening the crosslinking bonds of the hydrogel under acidic conditions (Table 2). By targeting the mildly acidic pH of the TME, these hydrogels can effectively concentrate drugs at tumor sites and help reduce systemic side effects. However, the sensitivity of some hydrogels to pH changes is a critical challenge. Some of the drug release curves exhibited significant differences with physiological conditions (pH around 7.4) only when the pH value decreased to 4.0, or even lower [52,53,54,55], which is much lower than the typical pH range of the TME (pH around 6.5 to 7.0). Such limitations may hinder their effectiveness, as the pH at most tumor sites may not be sufficiently low to induce drug release, potentially compromising therapeutic outcomes or causing off-target effects.

**Table 2:** pH-sensitive hydrogels for DDSs.

Loading Drugs	Polymers	DDS Mechanism	Drug Release pH	Degradation and Stability	Sterilization Method	In Vitro/In Vivo Analysis	Reference
DOX	Nitrogen-doped carbon quantum dots and hydroxyapatite	Disintegration of the Schiff base bond and dissolution of hydroxyapatite in acidic conditions	6.5–4.0	/	The introduction of HA by either chemical bonding or physical incorporation can result in hydrogels with enhanced antimicrobial activity	In vitro	[56]
DOX	N-carboxyethyl chitosan (CEC) and Di benzaldehyde-terminated poly (ethylene glycol) (PEGDA)	Disintegration of the Schiff base bond	6.8–4.0	Around 30% mass loss in PBS 7.4 and 50% mass loss in PBS 5.5 after 200 h	/	In vitro	[51]
DOX	Sericin, rice bran albumin, and gellan gum	Degradation in the ester bond and increase in the swelling ratio of the hydrogel	5.0–4.0	/	/	In vitro	[53]
Prospidine	Dextran phosphate	Diffusion of the cytostatic bond and the destruction of the polymer	4.0–1.2	100% mass loss after 30 days in PBS	/	In vitro and in vivo	[54]
DOX	Glycol chitosan–Pluronic F127 and α-CD	Dissociation of the hydrogel	5.0	100% mass loss after 220 to 260 h in PBS	/	In vitro and in vivo	[55]
Bortezomib	A ‘ABA’ triblock copolymer of phenylboronic acid-functionalized polycarbonate/poly (ethylene glycol)	Dissociations of boronated ester bond	5.8	At pH 7.4, the BTZ release from the micelle/hydrogel composite remained low at 7%, an acidic environment, ∼85% of BTZ was released over 9 days	/	In vitro and in vivo	[57]
DOX	Dextran-based nanogel	Disintegration of the Schiff base bond	5.0–2.0	/	/	In vitro	[58]
DOX	Carboxymethyl chitosan	Hydrolysis of ortho ester bond	6.5–5.0	Around 30% mass loss after 300 h in PBS 7.4 and around 40% mass loss after 300 h in PBS 5.0	/	In vitro	[59]
Gemcitabine, paclitaxel (PTX)	OE peptide (VKVKVOVK-VDPPT-KVEVKVKV-NH_2_)	Disruption of the 3D network of peptide from beta-sheet to random coil	5.8	/	/	In vitro and in vivo	[60]

Drug release pH: the pH condition used to measure the drug release profile from the DDS. DDS, drug delivery system; DOX, doxorubicin.

In contrast to pH-responsive hydrogels, thermosensitive hydrogels represent a class of physically stimuli-responsive hydrogels, designed with a particular focus on injectability. These hydrogels remain in a liquid state before injection and undergo gelation to the solid state at physiological temperatures (37 °C) [61,62,63,64]. This property allows thermosensitive hydrogels to be directly injected into tumor sites, where they provide sustained drug release. The application of these hydrogels minimizes the toxicity caused by drug diffusion and, more importantly, avoids the extensive tissue damage caused by surgical implantation. Additionally, the swelling and collapsing processes of thermosensitive polymer networks near their critical temperature have been used as a mechanism for drug delivery [65] due to the fact that therapeutic agents are commonly encapsulated within hydrogels, which results in a drug release profile governed by constrained diffusion of the agents through hydrogel networks [66].

Poly (N-isopropylacrylamide) (PNIPAAm) is one of the most commonly used polymers for preparing thermosensitive hydrogels. The sol–gel transition is driven by reversible changes between the hydrated and dehydrated states of PNIPAAm as the temperature crosses its lower critical solution temperature (LCST), typically below 37 °C [67]. Liu et al. developed an alginate-g-poly (N-isopropylacrylamide) copolymer system by conjugating PNIPAAm to alginate. This system remains dissolved in water or phosphate-buffered saline buffer at room temperature but self-assembles into a hydrogel when heated to body temperature (37 °C). DOX loaded onto a hydrogel exhibited sustained drug release and achieved significant antitumor effects against multidrug-resistant AT3B-1 cells [61]. Similarly, Luo et al. synthesized a PNIPAAm-coacrylic acid-g-F68 copolymer hydrogel for triptolide delivery. This hydrogel maintained a liquid state at room temperature and showed a marked increase in storage modulus when the temperature exceeded 35 °C. The antitumor agent, triptolide, was encapsulated in nanomicelles at room temperature and released intratumorally after injection. In animal models, the hydrogel exhibited superior antitumor efficacy and low toxicity compared with that of free drug administration [62].

In addition to the sol–gel translation behavior of thermosensitive hydrogels, the swelling and shrinking properties of hydrogels at their LCST have been widely used to control drug release [65,68,69]. For instance, PNIPAAm-based hydrogels typically exhibit LCST below 37 °C, collapsing and shrinking above the LCST while swelling and expanding below the LCST. This reversible swelling and shrinking behavior can be used in DDSs to control drug release profiles [70]. Lei et al. further advanced the design of thermosensitive hydrogels by developing a near-infrared (NIR)-triggered hydrogel for on-demand drug delivery. This thermosensitive chitosan (chitosan-g-PNIPAAm) polymer was synthesized by grafting PNIPAAm onto chitosan (chitosan-g-PNIPAAm) via free-radical copolymerization, followed by UV-induced crosslinking with methacryloyl groups. The resulting composite hydrogels exhibited significant shrinkage at elevated temperatures (45 °C). By incorporating photothermal carbon, the hydrogel could trigger the shrinkage of polymer networks upon NIR irradiation and thus release the loaded drug [71]. Although the composite hydrogels exhibited NIR-triggered behavior, the burst release profile of DOX following each exposure to 45 °C may negatively impact treatment outcomes, and the lack of detailed experimental data limits the evaluation of its clinical applicability. A critical mechanism underlying drug release control via the LCST is that below the LCST, the swelling of the hydrogel increases the diffusion distance for the encapsulated drugs, thus slowing their release. Conversely, above the LCST, the hydrogel network collapses, reducing diffusion resistance and accelerating drug release [65,70]. Therefore, it is essential to clarify the diffusion rate of drugs at various environmental temperatures because high environmental temperatures may accelerate drug release, potentially leading to misleading conclusions regarding hydrogel performance. A detailed investigation of these factors is crucial for optimizing LCST-based hydrogel systems for clinical use.

### 2.2. Cargo-Based Drug Delivery Hydrogels

The properties of hydrogels can be designed for DDSs using different materials; however, the encapsulation efficiency and release rates of drugs from hydrogels are influenced by multiple factors, even when using the same hydrogel matrix. Properties, such as hydrophilicity or hydrophobicity [72], electrostatic interactions [73], and molecular weight [74] significantly affect the drug delivery efficiency and antitumor efficacy. Consequently, the design and preparation of hydrogel-based DDSs must consider the physicochemical properties of the drugs and their specific delivery environment.

Reportedly, over 40% of marketed drugs exhibit poor water solubility, and approximately 60% of the compounds emerging from pharmaceutical research laboratories are classified as insoluble [75]. Among the challenges of hydrogel-based DDSs, the delivery of hydrophobic drugs via high-water-content hydrogels remains one of the most significant obstacles. Poor drug encapsulation efficiency and a low release rate often result in reduced bioavailability and therapeutic outcomes [76,77].

Paclitaxel (PTX), a widely used first-line antitumor agent with broad-spectrum antitumor effects, addresses these challenges. PTX functions as an anti-microtubule agent, binding specifically to the β-subunit of tubulin to inhibit microtubule disassembly, thereby arresting mitosis and suppressing tumor proliferation. In contrast, DOX hydrochloride (DOX · HCl), a water-soluble anthracycline, can bond to nucleic acids and form complexes with DNA by intercalating to base pairs to inhibit the function of topoisomerase II activity and then present antimitotic and cytotoxic effects. Owing to the hydrophilic and hydrophobic differences between these drugs, their release behaviors in the same DDS are markedly different. For example, in a study by Xu et al., the cumulative release of PTX from a hydrogel was only approximately 10% after 24 h, whereas DOX showed a much faster release and resulted in higher cytotoxicity against tumors than PTX after 48 h of culture. Conversely, the inhibition of cell proliferation by PTX was notably weaker even after 72 h, likely because of its low drug release rate [76]. Similarly, docetaxel (DOC), another taxane derivative with potent antitumor effects, is commercially available only as an intravenous formulation owing to its poor water solubility [78]. To address these limitations, strategies, such as combining hydrogels with liposomes, NPs, or microspheres, are commonly used as delivery tools for hydrophobic drugs. Furthermore, modifying the water solubility of drugs is commonly used (Table 3).

Amphiphilic polymers are particularly advantageous for delivering hydrophobic drugs. Their ability to self-assemble via hydrophobic interactions facilitates hydrogel formation and enhances drug encapsulation within the hydrogel. Rezazadeh et al. encapsulated PTX into mixed polymeric micelles composed of PF127 and tocopherol polyethylene glycol 1000 succinate (TPGS), which were subsequently incorporated into a hyaluronic acid (HA)-based hydrogel for the co-delivery of PTX and DOX. TPGS, with its amphiphilic structure, formed micelles in aqueous media and achieved a PTX entrapment efficiency of approximately 51%. Although the initial PTX loading affected the release rate, more than 50% of the drug was released from the hydrogel within 60 h, demonstrating efficient drug loading and release capabilities [79]. Although the study did not directly evaluate the antitumor efficacy of the hydrogel, subsequent in vitro and in vivo studies of similar DDSs demonstrated significant therapeutic effects [80].

The host–guest effect of cyclodextrin (CD)–drug complexes has been extensively explored as a strategy to improve the water solubility of hydrophobic drugs. CDs are cyclic oligosaccharides composed of D-glucopyranoside units linked via alpha-1,4 glycosidic bonds. The most commonly used CDs are alpha-CDs, beta-CDs, and gamma-CDs, which consist of six, seven, and eight glucose repeating units, respectively. The arrangement of these molecular structures forms a hydrophobic inner cavity and hydrophilic exterior [81], enabling CDs to encapsulate hydrophobic guest molecules within the cavity, thereby enhancing their physicochemical properties and aqueous solubility [82,83,84].

Nieto et al. used a host–guest reaction to load PTX into beta-CDs to form PTX: beta-CD inclusion complexes, which were subsequently encapsulated within a gellan gum hydrogel. The hydrogel was synthesized in different buffer systems (acetate and phosphate) and crosslinked with varying concentrations of L-cysteine using the crosslinker EDC/NHS to provide redox-responsive properties to glutathione (GSH). In drug release studies, approximately 50% of PTX was released from the hydrogel within 50 h under physiological conditions. Furthermore, the presence of L-cysteine and GSH allowed for adjustable drug release rates, demonstrating promising control over drug delivery and significant antitumor efficacy in vitro [85]. In another study, Ma et al. developed a beta-CD/camptothecin inclusion complex that was incorporated into folic acid hydrogels. The folic acid was chemically modified with NaOH and then crosslinked with ZnCl_2_ using a metal-ion-induced gelation method. The prepared CPT-beta-CD complex was added to the folic acid solution to form drug-loaded hydrogels [86]. This system leverages the selective affinity of folic acid for tumor cells, enhancing the targeting and delivery efficiency of CPT while mitigating off-target effects.

Notably, preclinical studies have demonstrated that CD–drug inclusion complexes often exhibit superior therapeutic performance compared with that of free drug administration [87,88,89]. However, most CD-based formulations are administered intravenously rather than incorporated into hydrogels for local drug delivery. Intravenous administration typically results in uncontrolled drug release, leading to systemic drug accumulation in non-target tissues and organs, which increases the risk of toxicity [90,91]. In contrast, combining CD–drug complexes with hydrogels provides an opportunity to leverage the sustained and localized release characteristics of hydrogels. This approach can enhance antitumor efficacy while minimizing systemic toxicity, particularly for hydrophobic drugs that pose substantial formulation challenges.

Furthermore, the application of CDs in local drug delivery commonly involves the formation of host–guest supramolecular hydrogels rather than the direct modification of drug properties. These supramolecular hydrogels have shown substantial potential for tumor therapy by providing tunable release kinetics, responsive behavior, and enhanced mechanical stability [92,93,94]. Combining drug complexes with hydrogel systems may optimize their therapeutic performance and clinical utility.

**Table 3:** Hydrogels for hydrophobic drug delivery.

Hydrophobic Drugs	Materials to Synthesize Hydrogels	Drug-Loading Mechanism	Drug Entrapment Efficiency	Drug-Loading Efficiency	Degradation and Stability	Sterilization Method	In Vitro/In Vivo Analysis	References
PTX	Mixed polymeric micelle composed of PF127, tocopherol polyethylene glycol 1000 succinate (TPGS), and hyaluronic acid (HA)	Micelle formation by hydrophobic interaction	~51%	~12%	Hydrogel maintained 75% of its original weight over 5 days at 37 °C, low viscosity at 4 °C converted to a semisolid upon heating to 35 °C	/	[76] In vitro [77] In vitro and in vivo	[79,80]
PTX	Gellan gum	Formed CD: drug complex	/	/	/	Sterilized by UV radiation	In vitro	[85]
PTX	PEGylated star polymer and PNIPAAm	Formed CD: drug complex	~90%	~3%	Complex nanoparticles experienced fast size broadening under physiological salt conditions (150 mM) within 30 min	/	In vitro and in vivo	[95]
PTX	Gellan gum modified by prednisolone	Hydrophobic interaction by prednisolone	~40%	/	Good stability	/	In vitro	[96]
PTX	IC1-R peptide	Beta-folded structure formation by hydrophobic interaction	~98%	/	Parameters set to a frequency of 0.1–100 rad/s, a sheer force of 1%, and a test time of 15 min. Fixed frequency of 6.28 rad/s, 0–5 min shear force set to 1%, and 5–7 min set to 100%. The shear force was set to 1% at 7–37 min, and the process was repeated at 37–70 min	Filtration via 0.22 μm filter	In vitro and in vivo	[97]
PTX, epirubicin	HA and poly (e-caprolactone)-poly (ethylene glycol)-poly-(e-caprolactone) (PCL-PEG-PCL)	Loading PTX to PCL-PEG-PCL nanoparticles	~90%	~10%	The HA-Gel embedded subcutaneously in mice gradually degraded 3 days post-implantation, and the extent of degradation was significant on day 5. Almost no residual hydrogel on day 9	/	In vitro and in vivo	[98]
PTX	PEG-PCL-PEG/DDP + MPEG-PCL/PTX	Loading PTX by monomethoxy PEG-PCL	~99%	~4%	When the temperature exceeded 25 °C, the samples changed from a liquid to an elastic gel-like substance at the crossover point of G and G; the composite’s viscosity was the strongest at about 43 °C	The prepared PDMP hydrogel composite was sterilized using C_O_-60 (20 Gy) before injection	In vitro and in vivo	[99]
Curcumin	Thiolated chitosan	Encapsulated by liposomes	~88%	~4%	[2] The deformation loss ratio was 20.06% (CSSH Gel), 23.92% (100 μM), 20.95% (150 μM), and 17.95 % (200 μM) after five cycles of compression [19]. The best compressive performance is at 5%, the compressive modulus of 5% is 27.44 kPa, and the maximum compressive strength is 32 kPa	/	In vitro and in vivo	[2,19]
Curcumin	Peptide (MAX8)	Beta-hairpin structure formation by hydrophobic interaction	/	/	Stable colloidal dispersion system and good mechanical properties	/	In vivo	[100]
Curcumin	Polyethylene glycol (PEG) and polycaprolactone (PCL) polymer PCL-PEG-PCL	Hydrophobic interaction by hydrophobic PCL	47~74%	2~9%	/	/	In vitro	[101]
Curcumin	Alginate/chitosan hydrogel microparticles	Loading curcumin by hyaluronic acid/zein nanoparticles	~70%	/	Cur/MPS in simulated gastric fluid (SGF) (pH 2) has no obvious change; NMPs can decompose rapidly in colonic fluid	/	In vitro and in vivo	[102]
Camptothecin (CPT)	Pluronic F127 and alpha-CDs	Micelle formation by hydrophobic interaction	/	/	/	Filtration via 0.45 μm filter	In vitro and in vivo	[92]
CPT	Folic acid and beta-CDs	Formed CD: drug complex	/	/	Good rheological mechanical property and thermodynamic stability	/	In vitro	[86]
Triptolide	PNIPAm-g-pluronic F68	Micelle formation by hydrophobic interaction	~84%	~5%	0.45 mg/kg TPL-equivalent dose three times over 14 days in 4T1 tumor-bearing mice	Filtration via 0.45 μm filter	In vitro and in vivo	[62]
DOX (DOX·HCl deprotonated at pH 9.6)	PEG-b-PCL and alpha-CDs	Micelle formation by hydrophobic interaction	72~74%	~15%	/	/	In vitro	[103]
DOC	Mixed polymeric micelle composed of PF127, PL121, and hyaluronic acid (HA)	Micelle formation by hydrophobic interaction	~99%	~2%	/	/	In vivo	[104]

DOC, docetaxel; DOX, doxorubicin; PTX, paclitaxel.

In addition to hydrophobic interactions and host–guest mechanisms, other advanced tools, such as NPs and liposomes, are commonly used in hydrogel-based DDSs [105,106]. Compared with the individual use of these tools, integration with high-water-content hydrogels for in situ delivery can provide dual benefits. The hydrogels enable localized and controlled drug release and simultaneously serve as a postoperative filler, offering additional benefits, such as promoting tissue regeneration or reducing inflammation at surgical sites.

**Figure 2:**
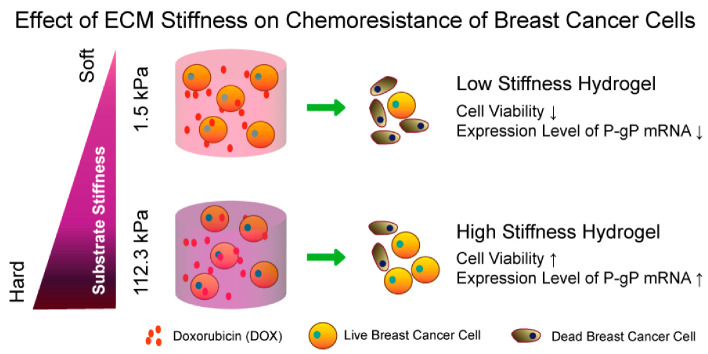
Extracellular matrix (ECM) stiffness affects chemoresistance of breast cancer cells to doxorubicin. Following encapsulation and culture in hydrogels, both cell viability and P-gp mRNA expression were found to decrease in the soft hydrogel, whereas they increased in the hard hydrogel. Copyright: Guoping Chen. There is no copyright issue [108].

Compared with free drug administration, the application of hydrogels is expected to fulfill at least one of the following three primary functions: (1) mitigating burst release to extend the therapeutic window and improve treatment efficacy; (2) minimizing systemic toxicity by reducing the drug dosage or enhancing local drug accumulation, thereby improving the survival curve; and (3) addressing drug incompatibility issues through spatiotemporal control of drug release. The first two functions are easily understood; however, the third requires an understanding of the complexity of tumor therapy. It is highly relevant to the common properties of tumors, including inherently heterogeneous and diverse genetic, epigenetic, and phenotypic variations that enable them to adapt and develop resistance to single-agent therapies. Moreover, not only can cancer cells develop drug resistance [107], but changes in the TMEs can contribute to single-drug resistance, thus leading to treatment failure (Figure 2) [108,109]. Therefore, combination therapy has been widely adopted for the treatment of various tumors in clinical settings. Established clinical protocols, such as FOLFOX (folinic acid, fluorouracil, and oxaliplatin) for colorectal cancer (CRC) and CHOP (cyclophosphamide, doxorubicin, vincristine, and prednisone) for non-Hodgkin lymphoma, exemplify the effectiveness of multi-agent regimens that significantly improve patient outcomes [110,111].

Despite its advantages, combination therapy presents challenges, such as increased complexity in pharmacokinetic and pharmacodynamic interactions, heightened risk of overlapping toxicities, and the need for precise dose optimization to prevent adverse effects [112,113]. Therefore, the development of advanced DDSs, including hydrogel-based platforms, has become popular for ensuring the controlled and targeted release of therapeutic agents in combination regimens.

### 2.3. Drug Delivery Hydrogels for Pathological Characteristics of Tumors

The distinct TME can influence drug delivery efficiency and is increasingly recognized as a unique characteristic that can be used in the design of DDSs, such as hypoxia, acidity, ultrahigh GSH levels, and the overexpression of specific enzymes [114]. Another important aspect is tumor heterogeneity, which also affects drug delivery and treatment efficiency yet remains an unresolved challenge. Heterogeneity refers to the various genetic and non-genetic phenotypes that emerge during tumorigenesis, progression, and treatment, as well as interpatient variability, even within the same tumor type [115,116]. Tumor vasculature is characterized by chaotic organization, high permeability, and substantial heterogeneity [117]. Additionally, cancer genotypes can vary across different microenvironments, possibly because of the different distributions of cytokines around tumor sites [118]. Moreover, significant spatial discordance in programmed death ligand 1 (PD-L1) expression has been observed between primary tumors and lymph node metastases in surgically resected gastroesophageal adenocarcinoma [119]. The heterogeneity of tumors not only reduces drug delivery efficiency by the disorganized vasculature [120] but also contributes to treatment failure through the emergence of drug-resistant cells and metastasis [121]. It also results in conflicting therapeutic outcomes and the unpredictability of clinical prognosis [119]. In single-agent DDSs, tumor heterogeneity leads to difficulties in precise targeted therapy [122], thereby compromising the effectiveness of hydrogel-based DDSs and diminishing therapeutic returns. Therefore, the development of combination therapy strategies using hydrogels has become a prevailing direction in efforts to enhance treatment efficacy [123,124,125].

#### 2.3.1. DDSs of Cytotoxic Drugs

DOX is a model drug widely used in DDSs and acts via multiple mechanisms [126]. Despite its effectiveness, the non-negligible cardiotoxicity of both dose- and schedule-dependent DOX significantly limits its clinical application to a substantial extent [127]. Additionally, short-term treatment with DOX demonstrated a low antitumor effect [128]; therefore, long-term treatment is recommended but is associated with cumulative toxicity, followed by reduced therapeutic utility and eventual exhaustion of antitumor effectiveness, as evidenced by tumor regrowth typically observed at approximately 20 days post-administration in most in vivo experiments [129,130,131]. Collectively, efforts to mitigate the systemic toxicity of DOX, such as reduction or altered administration schedules, show potential, but often compromise the required therapeutic efficacy. Conversely, high-dose administration enhances antitumor outcomes but exacerbates toxicity [127,130,132]. To balance these competing factors, the combination of DOX with other antineoplastic agents has emerged as a promising strategy, particularly for hydrogel-based DDSs (Table 4).

Zhao et al. developed an injectable hydrogel system composed of glycol chitosan (GC) and amphiphilic hydrogels (OHC-PEO-PPO-PEO-CHO) to co-deliver DOX and PTX. The system showed a burst release of DOX and a sustained release of PTX owing to differences in their water solubility. Although the combination of DOX and PTX did not yield significantly enhanced antitumor efficiency, which is likely due to the asynchronous release profiles of the two drugs, the toxicity profile was not exacerbated by the prolonged survival time in comparison with a single DDS [133]. In another study, PTX and DOX were loaded with PECT and PEPF, respectively, and NPs prepared using nanoprecipitation technology were incorporated into hydrogels. A hydrogel containing drug-loaded NPs was then synthesized and achieved better antitumor efficacy and reduced systemic toxicity compared with free drug administration [134].

In addition to DOX, cisplatin is another important chemotherapeutic agent used to treat various malignancies, including sarcoma, carcinoma, and lymphoma. Cisplatin, or cis-diamminedichloroplatinum (II) (CDDP), is a platinum-based chemotherapeutic agent that exerts antitumor effects by forming DNA crosslinks, thereby disrupting DNA synthesis and transcription to induce tumor cell apoptosis. Despite its antitumor effects, the widespread uptake of CDDP by both normal and tumor cells via ubiquitously expressed transporters results in significant side effects, thereby limiting its application [135]. For instance, the specific expression of organic cation transporter-2 (OCT2) in the kidneys may explain the nephrotoxicity of CDDP [136,137]. The high water solubility and rapid burst release of CDDP at the time of administration further contribute to its systemic toxicity and suboptimal antitumor efficacy. Hydrogel-based DDSs have been used to address these challenges by retarding the drug release rate and enabling the localized delivery of drugs to tumor sites, thus improving therapeutic outcomes while avoiding off-target effects [138,139,140]. For example, a study by Li et al. demonstrated that incorporating CDDP into thermosensitive hydrogels not only provided controlled and sustained release but also enhanced tumor suppression while significantly reducing renal toxicity in animal models [141].

As for the co-administration of CDDP and other therapeutic agents, Chen et al. developed a co-delivery system using a biodegradable temperature-sensitive hydrogel (PDLLA-PEG-PDLLA, PLEL) for combination chemotherapy of gastric tumors. The thermosensitive hydrogel not only enhanced local combination therapy with 5-fluorouracil (5-FU) and CDDP but also demonstrated significantly better antitumor efficacy than that of the free administration of 5-FU and CDDP. Moreover, the hydrogel promoted the local accumulation of 5-FU and CDDP. Moreover, the hydrogel system promotes the localized accumulation of drugs, reduces systemic exposure, and extends the overall survival time of treated animals with minimal tissue damage and reduced systemic toxicity [138]. Similarly, Shen et al. investigated a self-assembled poly (ethylene glycol) (PEG)/polyester copolymer hydrogel for the co-delivery of CDDP and PTX to ovarian tumor models. This hydrogel-based DDS demonstrated low systemic toxicity, as evidenced by minimal weight loss in the experimental animals. Although PTX showed a slower release rate than that of CDDP, both drugs maintained a steady release profile throughout the study, contributing to enhanced antitumor efficacy through sustained therapeutic concentrations at the tumor site [139].

To further enhance antitumor efficiency, other chemotherapeutic agents with distinct mechanisms, such as topotecan, SN-38, and 5-FU, have also been explored in hydrogel carrier systems [142,143,144,145]. These studies highlighted the multiple advantages of hydrogel-based DDSs for combination therapy. First, hydrogels mitigate burst release effects and reduce the systemic toxicity associated with free drug administration. By tailoring the hydrophilic and hydrophobic properties of the hydrogel matrix, controlled and localized drug delivery can be achieved, thereby improving the therapeutic index. Second, the differential solubility of the co-administered drugs can be leveraged to regulate their overall concentrations at the target site, ensuring sustained exposure and enhanced synergistic effects.

Additionally, hydrogels enable the sequential or simultaneous delivery of multiple drugs, facilitating combination therapies that synergistically exploit distinct antitumor mechanisms. This approach is particularly effective for overcoming drug resistance because it disrupts adaptive cellular responses and maintains therapeutic pressure. Furthermore, hydrogels can bridge pharmacokinetic gaps in drug metabolism cycles by providing controlled release and preventing therapeutic windows in which tumor cells can recover or proliferate.

By co-delivering hydrophilic and hydrophobic drugs on a single platform, hydrogel systems can maximize the efficacy of chemotherapeutic agents while minimizing off-target effects. These systems exemplify a powerful strategy for achieving precise and effective combination chemotherapy, paving the way for personalized and tolerable cancer treatments.

**Table 4:** Combinations of different antineoplastic agents.

Tumor	Drugs	Materials to Synthesize Hydrogels	Effects	Reference
Melanoma	DOX and PTX	Glycol chitosan and benzaldehyde-terminated polymer	High antitumor efficacy and safety	[133]
Colon carcinoma	DOX and DOC	Micelles by PL121, PF127, and HA	High antitumor efficacy and safety	[104]
Bladder carcinoma	DOX and CDDP	PEG-b-PCL and alpha-CDs	Controllable drug release and injectable ability	[103]
Hepatoma	DOX and Curcumin	PCL-PEG-PCL	High antitumor efficacy and safety	[101]
Lung tumor	PTX and CDDP	PEG-PCL-PEG and PMEG-PCL	High antitumor efficacy, controllable drug release, and safety	[99]
Ovarian tumor	PTX and CDDP	Bi-mPEG-PLGA-Pt (IV)	High antitumor efficacy and safety	[139]
Breast tumor	PTX and Epirubicin (EPI)	PCL-PEG-PCL and HA	High antitumor efficacy, controllable drug release, and safety	[98]
Breast tumor	PTX and Gemcitabine (GEM)	OE peptide hydrogel	High antitumor efficacy	[60]
Breast tumor	PTX and Honokiol	PLGA-PEG-PLGA	High antitumor efficacy and safety	[146]
Ovarian tumor	PTX, Rapamaycin, LS301	PLGA-b-PEG-b-PLGA	High antitumor efficacy	[147]
Colorectal peritoneal carcinomatosis	CDDP and 5-FU	Ring-opening copolymerization to synthesize ε-CL and PEG and chitosan	High antitumor efficacy and safety	[145]
Gastric tumor	CDDP and 5-FU	PDLLA-PEG-PDLLA	High antitumor efficacy, safety, and high efficacy to inhibit tumor recurrence	[138]
Pancreatic tumor	CDDP and GEM	PDLLA-PEG-PDLLA	High antitumor efficacy	[148]
Colorectal tumor	Oxaliplatin and Hesperetin	Cationic Okra gum	High cytotoxicity	[149]
Brain tumor	Carmustine and Curcumin	PCL-PEG	High efficacy to inhibit tumor recurrence	[150]

DOC, docetaxel; DOX, doxorubicin; HA, hyaluronic acid; PTX, paclitaxel.

#### 2.3.2. DDSs of Anti-Angiogenesis Drugs

Generally, tumor growth is characterized by uncontrolled proliferation, which leads to a high metabolic demand, rapid nutrient consumption, and increased production of waste products. Unlike blood cancers and other nonsolid tumors, the growth of solid tumors generally requires vascularized connective tissue stroma to sustain expansion beyond their minimum size [151]. It is widely accepted that the vasculature in tumors is chaotic with high permeability and heterogeneity compared with that in normal tissues [152,153]. As tumors rely on angiogenesis for their continued growth and metastasis, angiogenesis therapy has been recognized as a critical target in tumor treatment, promoting the development of numerous anti-angiogenic agents, such as bevacizumab, cediranib, axitinib, and regorafenib [154,155]. Other anti-angiogenic agents, such as inhibitors of tyrosine kinase receptors, including sunitinib, sorafenib, and pazopanib, have found broad clinical applications [156]. Anti-angiogenic strategies transiently normalize tumor vasculature and enhance the delivery and efficacy of chemotherapeutic agents [157,158]. Furthermore, targeting tumor angiogenesis facilitates metronomic chemotherapy, a regimen of chronic, low-dose drug administration aimed at preventing angiogenesis and inducing sustained antitumor effects [159]. According to these theories, the combination of anti-angiogenesis and chemotherapy has become a widely adopted therapeutic model [160].

To address the toxicity and limited efficiency of anti-angiogenesis therapies, hydrogels have been explored for localized drug delivery and the controlled release of the drugs. Li et al. used a classic injectable thermosensitive PLGA-PEG-PLGA hydrogel to co-deliver the vascular disruptive agents, combretastatin A-4 (CA4) disodium phosphate (CA-4DP) and epirubicin. Combination therapy not only exhibited superior antitumor effects compared with that of single-drug administration but also prolonged the survival of treated animals [161].

CA4P is a water-soluble prodrug of CA4, which demonstrates robust antitumor efficacy, particularly in combination therapies for platinum-resistant ovarian tumors. This is a mechanism that induces vascular congestion and reduces tumor blood flow by changing the endothelial cell morphology to achieve treatment outcomes [162]. However, the vascular effects of CA4 can negatively affect the efficacy of concurrently administered drugs, which necessitates high requirements for controlling the sequence and timing of drug release [163]. Wei et al. addressed this challenge by designing a polypeptide-based hydrogel capable of sequentially releasing CA4 and DOX. By utilizing the distinct hydrophobic properties of these drugs, their hydrogel facilitated the faster release of DOX and the slower release of CA4, minimizing potential negative interactions and optimizing therapeutic efficacy [63]. Conversely, Wang et al. reported a different release profile using an injectable PLGA-PEG-PLGA triblock polymer hydrogel. They found that CA4P, which is more hydrophilic than DOX, was released at a faster rate. Despite this reversed release sequence, combination therapy yielded superior antitumor effects compared with single-drug treatments, highlighting the versatility and potential of hydrogel-based co-delivery systems [164].

Regardless of the agent that should be released first, it is important to avoid drug interactions with anti-angiogenic and antineoplastic agents. Owing to the different mechanisms of anti-angiogenic and antineoplastic agents, the design and synthesis of hydrogels must be considered. In addition to enhancing the antitumor effect, avoiding high toxicity, preventing negative drug interactions, and the time-dependent induction of drug resistance by anti-angiogenic therapies [165,166] increase requirements for hydrogel-based DDSs. The preparation of smart hydrogels for the combination of antineoplastic and anti-angiogenic agents is urgently required.

#### 2.3.3. DDSs of Immune Checkpoint Inhibitors

Cancer treatment includes various strategies beyond conventional chemotherapy, such as surgery, radiotherapy, immunotherapy, and gene therapy. Combination therapy leverages the complementary mechanisms of different approaches to enhance efficacy, mitigate resistance, and improve overall outcomes. In the case of advanced non-small-cell lung cancer, the combination of immune checkpoint inhibitors with chemotherapy in a first-line treatment prolongs the survival of patients in phase III trials [167]. Hydrogel-based DDSs can facilitate the combination of cytotoxic drugs for cancer treatment and other treatment approaches by providing controllable and localized release of different therapeutic agents. As an example of chemotherapy combined with immunotherapy, Chao et al. developed an alginate-based hydrogel to deliver the chemotherapeutic agent oxaliplatin (OXA) and immune adjuvant imiquimod (R837) as a “cocktail” of chemoimmunotherapeutic composites. The composite was administered intratumorally to form a hydrogel for localized treatment. In addition, an immune checkpoint blockade antibody (anti-PD-1) was included in the injection solution to enhance local immune modulation. The combination of OXA, R837, and anti-PD-1 within the hydrogel matrix showed superior antitumor efficiency compared with that of the free administration of these agents [168]. In another study, a nanogel was designed for combined chemoimmunotherapy by crosslinking carboxymethyl chitosan-derived polymetformin loaded with DOX. This system efficiently inhibited tumor growth, as polymetformin reshaped the TME and DOX killed the tumor cells more effectively [169].

Owing to the complexity of immune responses, designing hydrogels for combining chemotherapy and immunotherapy often requires meticulous consideration of both drug release profiles and treatment sequences. Animal studies typically involve multifaceted validation to assess the additive or synergistic effects of therapeutic strategies. Sun et al. used immunogenic cell death (ICD) to develop a hydrogel capable of administering immune adjuvants to tumors in a controlled manner during each cycle of chemotherapy and radiotherapy. The hydrogel is designed to release immunotherapeutic factors in response to low doses of oxaliplatin and X-rays. This approach results in a marked synergistic effect between chemotherapy and immunotherapy. By avoiding the simultaneous administration of chemotherapy and immunotherapy, this design effectively reduces systemic toxicity. More importantly, because ICD is induced by the release of chemotherapeutic agents or radiation therapy, it leads to the generation of a robust immune response, which has demonstrated a potent therapeutic effect in animal models. Hydrogel-based DDSs have been extensively reported as a means of minimizing the adverse effects associated with systemic immunotherapy. These systems facilitate the controlled release of therapeutic agents, thereby improving both safety and efficacy [170].

**Figure 3:**
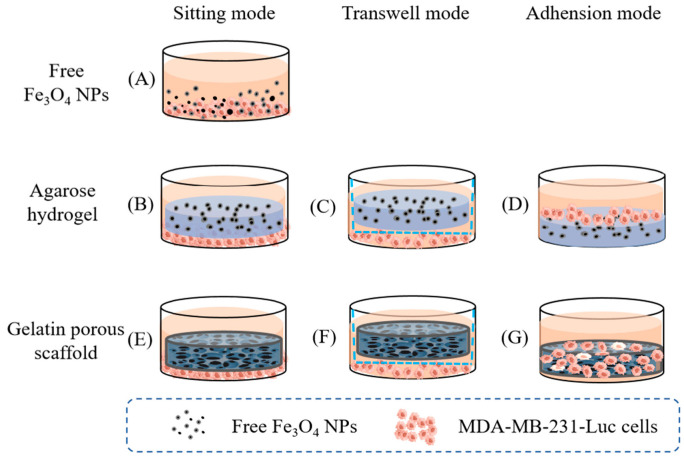
Anticancer experimental scheme of free Fe_3_O_4_ NPs (**A**), agarose/Fe_3_O_4_ hydrogels (**B**–**D**), and gelatin/Fe_3_O_4_ porous scaffolds (**E**–**G**). Three modes (sitting, Transwell, and adhesion modes) were used to simulate the cells near or far away from or directly adhered to the matrices. Copyright: Guoping Chen. There is no copyright issue [173].

From a fundamental perspective, one of the most valuable attributes of hydrogel-based DDSs in combination therapies is their ability to control drug release based on the distinct solubility profiles of the incorporated drugs. Engineering hydrogels with appropriate and tunable affinities for specific drugs may be critical to achieve optimal DDS performance. Other types of hydrogels, such as hydrophobic hydrogels [171], tough adhesive hydrogels [172], and specialized formulations, are also under investigation. Owing to their high elasticity, water content, plasticity, and tunable gelation properties, hydrogels represent an indispensable DDS platform with immense potential for research and clinical applications. Furthermore, hydrogels can be used to carry NPs and serve as fillers for treatments. Magnetic NPs exhibit stronger thermal effects in hydrogels than conventional scaffold materials (Figure 3) [173], making hydrogels not only effective DDSs but also potentially transformative as post-surgical fillers with enhanced therapeutic outcomes. This multifunctionality broadens the scope of hydrogel applications in oncology, highlighting its versatility as both a drug carrier and therapeutic material.

## 3. Methods for In Vitro and In Vivo Evaluations

### 3.1. Method for In Vitro Antitumor Effect Evaluation

**Figure 4:**
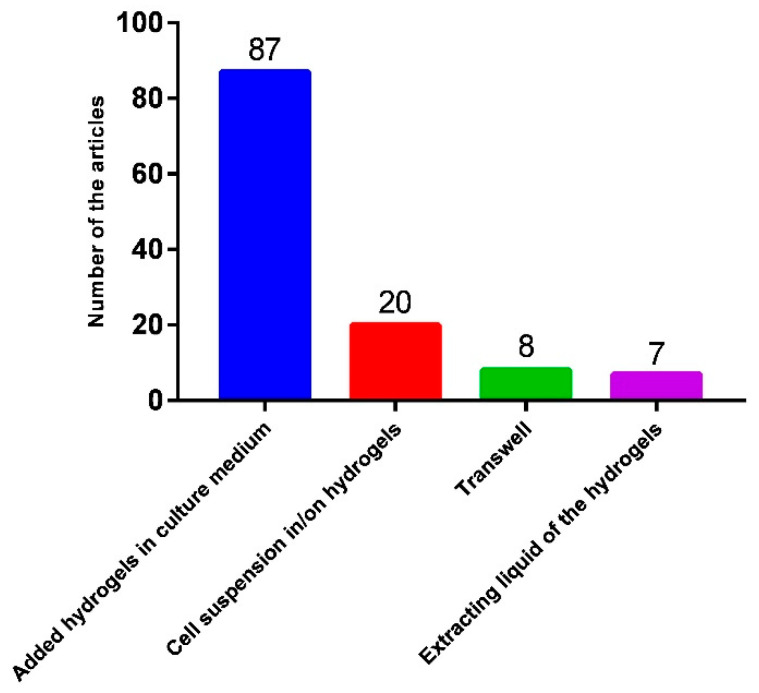
Summary of the methods used to analyze antitumor effects at the cellular level.

The evaluation of antitumor efficiency is critical for determining the applicability of drug-loaded hydrogels. Currently, four primary methods are commonly used to assess the cytotoxicity of drug-loaded hydrogels (Figure 4). The most widely used approach, reported in over 70% of the studies, involves treating cells with a medium containing hydrogel. Approximately 20% of the studies used a method in which cell suspensions were deposited directly into the hydrogel for co-culture. Among the 106 studies analyzed, six used hydrogel extracts for treatment and seven studies used Transwell systems to analyze cytotoxicity. Each of these methods have distinct advantages and limitations.

Adding hydrogels or their extracts directly to the culture medium is convenient, which is not different from methods that add hydrogel extracts with drugs into the culture medium and allow the simultaneous evaluation of hydrogel biocompatibility and cytotoxicity. However, these tests are typically conducted over a short period (48–72 h), which is considerably shorter than the extended duration of drug release observed in practical applications. Therefore, these methods may not accurately represent the actual antitumor efficacy of hydrogels. The method involving the deposition of cell suspensions on/into the hydrogels provides a closer approximation of the real-world applications of drug-loaded hydrogels. However, this approach inherently reflects the behavior of cells in direct contact with the hydrogel without considering the effects of remote DDSs. Moreover, drug diffusion from the hydrogel can significantly affect antitumor efficiency, necessitating the optimization of culture conditions, such as prolonging the incubation period, to better simulate in vivo drug release dynamics.

The Transwell assay focuses on assessing the cytotoxicity of drugs released from hydrogels. Although this provides valuable insights into drug delivery and release efficacy, it lacks the ability to directly evaluate hydrogel biocompatibility. The primary purpose of using a hydrogel-based DDS is to enhance the safety of drug administration and achieve localized drug concentrations; a comprehensive evaluation strategy is essential. It would be more reasonable to test the toxicity of the hydrogels and the optimal concentration of antitumor drugs.

### 3.2. Method for In Vivo Antitumor Effect Evaluation

The results of animal experiments for DDSs are more generalizable for clinical use than those of in vitro experiments. Although hydrogel-based DDSs have been extensively studied, some studies are still in the preclinical stage [174]. To reveal the real effects of hydrogel-based DDSs, in vivo experiments are indispensable before preclinical analysis.

**Figure 5:**
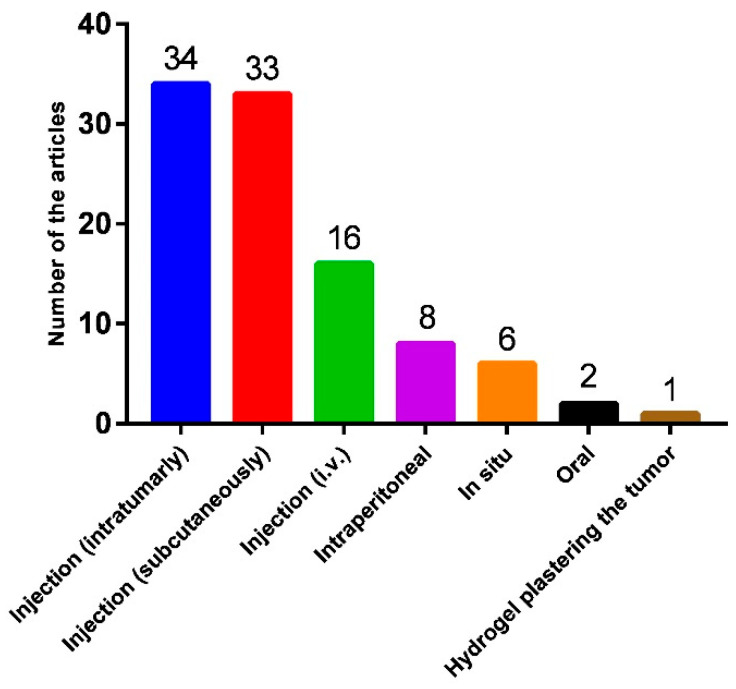
Summary of the methods used for animal experiments.

Numerous studies have reported the antitumor effect of hydrogels in vivo, with some focusing on their efficacy in animal models of recurrence. Unlike free drug administration, the dosage regimen of hydrogels typically involves intratumoral, peritumoral, or subcutaneous injections. These methods require tailoring of the rheological properties of hydrogels to meet the requirements of injectability and sol–gel transition under in vivo conditions (Figure 5).

Peritumoral or intratumoral injections maximize local drug concentrations and antitumor efficacy, minimize drug loss through the circulatory system, and reduce accumulation in non-target organs, thereby lowering systemic toxicity compared to intravenous injections [134,144,175,176].

In contrast, in situ administration is more frequently used in brain tumor research [177,178,179]. For brain tumor treatments, the blood–brain barrier presents a considerable challenge, reducing effective drug concentrations at the target site and potentially causing off-target effects and systemic toxicity [180,181]. Compared with NP-based DDSs, in situ injection is more important for analyzing the function and availability of hydrogel-based DDSs in brain tumors, and subcutaneous tumor models cannot replicate the challenges posed by the unique environment of the brain.

Moreover, for specific therapies, such as immunotherapy, hydrogel carriers have demonstrated higher efficacy than that of free drug administration [182]. This may be attributed to the rapid clearance or degradation of immune-related agents, such as antibodies, during free administration, which reduces their therapeutic activity [183]. The application of hydrogels mitigates this issue by protecting these agents and enabling their sustained release, thereby enhancing their efficacy.

In addition to a single method of administration, in most studies, researchers have used more than one experimental method to evaluate the treatment potential of hydrogel-based DDSs. Jin et al. investigated the tumor ablation effects of injectable peptide hydrogels co-delivering DOX and Melttin. Their study compared intratumoral, peritumoral, and in situ injection methods and demonstrated significant antitumor effects across all approaches using hydrogel-based DDSs. In a footpad model used to study lymphatic metastasis, the in situ injection of hydrogels also achieved effective tumor suppression [184]. For the same hydrogel material, intratumoral and peritumoral injections showed similar results, likely due to the close proximity of the injection sites to the tumor [104]. In various studies, intratumoral or peritumoral administration consistently exhibited higher antitumor efficacy than that of free drug administration via intravenous injection [134,175,185]. Even when tumor growth inhibition was comparable, localized administration methods resulted in lower systemic toxicity, as reflected by the reduced body weight loss in treated animals [176]. These findings underscore the significant advantages of hydrogel-based DDSs in improving therapeutic outcomes while minimizing adverse effects.

Despite the gradual comprehensive analysis of hydrogel-based DDSs, challenges remain in translating these systems into practical applications. One issue is the discrepancy between the administration methods used in the experiments and those applicable to preclinical models. For disease treatment, intravenous, intramuscular, and subcutaneous injections remain the most common routes, whereas intratumoral injection is primarily used in antitumor immunotherapy at the preclinical stage [186,187,188]. Hydrogel injection would involve more problems than single immune agent injection. Furthermore, the feasibility of injection imposes additional constraints on the selection of hydrogel materials.

Apart from administration approaches, the variability in animal experiments in various studies poses substantial challenges. Differences in tumor volume at the start of experiments and research-dependent injection sites for peritumoral administration make it difficult to precisely evaluate treatment efficiency. Therefore, it is necessary to develop standardized and reliable animal models and experimental protocols to facilitate accurate comparison and reproducibility in hydrogel-based DDS research.

Finally, the lack of attention to sterilization methods in many studies is another issue. Only a few studies provided detailed descriptions of the sterilization process [189]. Although some hydrogels incorporate antibacterial drugs [190] or are composed of inherently antibacterial materials [191], most studies have not described sterilization methods, particularly for long-term animal experiments. Although Schiff base interactions have antibacterial effects [192], programmed sterilization remains essential for both in vivo studies and further applications.

## 4. Challenges and Developments of Hydrogel-Based DDSs

### 4.1. Preclinical Evaluation and Clinical Trials

No matter how innovative or imaginative a concept may be, it ultimately needs to be translated into practical applications. Only when favorable therapeutic efficacy is demonstrated can a hydrogel-based system proceed to clinical trials and eventual implementation. Currently, the major limitations hindering the clinical translation of hydrogel-based DDSs include suboptimal therapeutic efficiency, safety concerns, and insufficient objective preclinical evaluations.

For hydrogel-based DDSs, the most critical factor is drug delivery efficiency, which directly affects treatment effectiveness. Macroscopically, this efficiency is influenced by two key factors: the design of the hydrogel and the biological characteristics of the tumor. In terms of the hydrogel design, the mesh size of the hydrogel polymers, interactions between drugs and hydrogels, and drug concentration significantly affect the overall DDS performance. These factors have been extensively discussed in previous reviews [66,193,194]. However, pathophysiological characteristics of tumors also play a crucial role in drug delivery efficiency and cannot be ignored. The development of DDSs that adapt to the TME, along with combination therapy-based delivery strategies, is a major research topic in tumor treatment research. Specifically, with the application of novel materials and the development of innovative antitumor agents, the integration of bioactive compounds with hydrogel polymers offers expanded possibilities for tumor therapy.

Hydrogels exhibit excellent biocompatibility, supporting their broad clinical potential. However, the introduction of chemical crosslinking agents and the degradation of hydrogel polymers may inadvertently trigger undesirable biological responses, leading to safety concerns regarding their use [195,196]. For example, the incorporation of crosslinkers, such as glutaraldehyde, alters macrophage polarization during the foreign body response. This response is typically characterized by ECM deposition around the implanted biomaterial, which can impair device function and induce chronic inflammation [196,197]. Moreover, the degradation products of certain synthetic hydrogels, such as polyesters, poly (glycolic acid), and poly (L-lactic acid), may generate low-molecular-weight byproducts with cytotoxic or immunogenic properties [198,199]. These byproducts can induce oxidative stress, promote pro-inflammatory cytokine release, or interfere with local tissue homeostasis, raising concerns regarding long-term biocompatibility. However, most degradation analyses are conducted over short durations and the characterization of the degradation products is often lacking (Table 1, Table 2 and Table 3). Therefore, understanding and controlling the immunotoxicology profiles of both crosslinking agents and degradation products is essential for the safe translation of hydrogel-based DDSs into clinical use.

Despite these uncertainties, progress in clinical trials continues to demonstrate the strong potential of hydrogel-based DDSs for clinical applications. Currently, a hydrogel developed for the effective release of fibroblast growth factor-binding protein 1 (FGFBP1) inhibitors to treat pancreatic adenocarcinoma is in progress at CNR Nanotec Lecce [200]. Additionally, a thermosensitive hydrogel for 5-FU delivery for CRC treatment is in the recruitment phase [201]. The FDA has approved the chemotherapy gel, Jelmyto, for the treatment of low-grade upper tract urothelial cancer. Jelmyto is a thermally responsive hydrogel used to deliver the cytotoxic chemotherapeutic agent mitomycin [202]. Another promising candidate, UGN-102, a reverse thermal hydrogel with a mechanism similar to that of Jelmyto, has been developed for the primary nonsurgical treatment of low-grade, intermediate-risk, non-muscle-invasive bladder cancer. It has shown a high complete response rate in Phase III trials and may serve as a potential alternative to surgery [203].

Notably, both Jelmyto and UGN-102 offer minimally invasive approaches to chemotherapy [203]. Other non-drug delivery methods using hydrogels, such as the rectal hydrogel spacer system SpaceOAR [204], achieve a therapeutic effect by injection into the site of action. In line with the clinical requirements, most in vivo experiments have focused on the development of injectable hydrogels (Figure 5). However, their degradation behavior and immunogenicity have attracted less attention. More importantly, infection has also been reported as one of the complications associated with hydrogel injection, whereas sterilization methods are rarely described in hydrogel preparation protocols (Table 1, Table 2 and Table 3). In summary, although several hydrogel-based DDS candidates have entered clinical trials [205,206], insufficient preclinical data hinders the clinical translation of many promising systems. Completing in vivo studies with robust and sufficient data, particularly regarding the degradation safety and preparation methods, would be substantially beneficial for accelerating the entry of hydrogel-based DDSs into clinical use.

### 4.2. Future Directions and Conclusions

In this review, we summarized the strategies and methods commonly used to prepare hydrogel-based DDSs for antitumor therapies. Unlike traditional administration, hydrogel-based DDSs not only avoid blast drug release but are also applicable for combination therapy design. Hydrogel-based DDSs represent a promising frontier in antitumor therapy owing to their unique properties, such as high water content, biocompatibility, tunable physical and chemical characteristics, and the ability to encapsulate a variety of therapeutic agents. These features make hydrogels well-suited for addressing key challenges in cancer treatment, including enhancing local drug concentrations, reducing systemic toxicity, and enabling controlled drug release. Despite significant progress, several avenues remain unexplored, providing opportunities for future research.

One critical area of advancement is the integration of smart and responsive hydrogel technologies. Hydrogels that respond to external stimuli, such as temperature, pH, and magnetic or electric fields, are increasingly being investigated for their potential to achieve spatiotemporal drug release. Future studies should focus on developing multi-responsive hydrogels capable of adapting to the dynamic TME, thereby improving the precision of drug delivery and therapeutic outcomes.

Another promising direction is the application of hydrogels based on the clinical demand for tumor treatment, such as the combination delivery of different antitumor drugs, the combination delivery of antitumor and anti-angiogenic agents, and the combination of chemotherapy and other therapeutics. These hybrid systems can leverage the advantages of each component to improve the targeting specificity and achieve multilevel drug release profiles. Additionally, because of their good biocompatibility and designable mechanical properties using different polymers, hydrogel systems can further broaden their application as postoperative fillers for tissue regeneration.

Despite rapid developments, several challenges need to be addressed for the translation of hydrogel-based DDSs from the laboratory to clinical settings. Biodegradability and biocompatibility must be meticulously evaluated to ensure that the hydrogel degradation products are nontoxic and do not elicit adverse immune responses. Furthermore, the scalability and reproducibility of hydrogel synthesis must be optimized for mass production under good manufacturing practice standards.

The versatility and adaptability of hydrogels make them well-positioned to play a pivotal role in personalized medicine. As research continues to reveal the complexities of tumor biology, hydrogel-based systems are expected to evolve into more sophisticated and effective tools for cancer therapy, with promising improvements and reduced treatment burdens for patients worldwide.
